# A comparative study of three CT and MRI registration algorithms in nasopharyngeal carcinoma

**DOI:** 10.1120/jacmp.v10i2.2906

**Published:** 2009-04-22

**Authors:** XiaoShen Wang, LongGen Li, ChaoSu Hu, JianJian Qiu, ZhiYong Xu, Yan Feng

**Affiliations:** ^1^ Department of Radiation Oncology, Cancer Hospital Fudan University Shanghai People's Republic of China 200032

**Keywords:** NPC, CT, MRI, image registration

## Abstract

Objective: To evaluate the image registration accuracy and efficiency of CT and MRI fusion using three algorithms in nasopharyngeal carcinoma (NPC). Methods and materials: Twelve sets of CT and MRI scans of 12 NPC patients were fused using three image registration algorithms, respectively: Mark‐and‐link, Interactive, and Normalized Mutual Information (NMI). Registration accuracy was evaluated by performing statistical analysis of the coordinate differences between CT and MR anatomical landmarks along the x‐, y‐ and z‐axes. The time required to complete the registration process using three algorithms was also recorded. One‐way ANOVA was used to analyze the difference of the three registration methods. Results: The mean time required for CT/MRI registration using the three different registration algorithms, mark‐and‐link, interactive, and NMI, was 6.25 min, 5.25 min, and 5.15 min, respectively. The mark‐and‐link method was more time consuming (F=8.74,p=0.001); however no statistical difference was found between the time required using interactive and NMI methods (p=0.77). Mean registration errors of the three methods along the x‐axis were 0.66 mm, 0.70 mm, and 0.68 mm, respectively (F=0.09,p=0.91). Along the y‐axis, the mean registration errors were 1.03 mm, 1.04 mm, and 1.03 mm, respectively (F=0.02,p=0.98). Along the z‐axis, they were 0.58 mm, 0.64 mm, and 0.56 mm, respectively (F=0.21,p=0.81).

Conclusions: All three registration algorithms, mark‐and‐link, interactive, and NMI, can provide accurate CT/MRI registration. However the mark‐and‐link method was most time consuming.

PACS number: 87.57.nj

## I. INTRODUCTION

Correct determination of tumor localization and extension is of major importance in radiation oncology. This is especially true from the perspective of modern radiotherapy (RT) techniques such as 3D conformal and intensity‐modulated radiotherapy (IMRT). These techniques offer the possibility of dose escalation and improved sparing of normal tissues. The precise delineation of gross tumor volume (GTV) is one of the quality assurance aspects that have to be dealt with when applying these techniques.^(1)^

Two imaging modalities, CT and MRI, have been utilized in outlining the GTV in nasopharyngeal carcinoma (NPC). CT is commonly used in three‐dimensional (3D) RT planning because it provides the superior spatial accuracy and electron density information necessary for heterogeneity corrections in dose calculation. A disadvantage of CT is, however, its poor soft‐tissue contrast. MRI, on the other hand, provides superior soft‐tissue contrast and visualization of tumor invasion of surrounding soft tissues. In addition, MRI provides images in nonaxial planes (sagittal and coronal), thus allowing better 3D representation of the tumor volume. However, MR imaging suffers from geometric distortion at the edges of the field of view and are susceptible of artifacts at interfaces between bone and air. Furthermore, it does not provide the intrinsic information on electron density, which thereby precludes its use as the sole imaging modality for treatment planning in NPC. An accurate image registration of CT and MRI scans is essential in treatment planning,^(^ ^2^ ^–^ ^4^ ^)^ because the complementary information contained in the two modalities can provide more accurate tumor definition.^(^ ^5^ ^–^ ^9^ ^)^

The use of commercially available registration software is growing rapidly in radiotherapy centers. A number of registration algorithms have been described as providing generally satisfactory results.^(^ ^10^ ^–^ ^16^ ^)^ Three registration methods are commonly used in our clinic: mark‐and‐link, interactive (AcQPlan 4.1, Philips Medical Systems, Bothell, WA), and Normalized Mutual Information (NMI) (ADAC Pinnacle^3^, ADAC Laboratories, Milpitas, CA).

In this paper, we describe our experience of CT and MRI registration in NPC using the above three methods, and compare the registration accuracy of the three algorithms.

## II. MATERIALS AND METHODS

### A. Patient Selection

In this study, we selected the image data sets of 12 NPC patients who received 3D conformal RT. Both planning CT and MR scans of the skull base, nasopharynx, and neck were acquired for all patients for the purpose of radiotherapy treatment planning.

### B. data Acquisition

**Figure 1:**
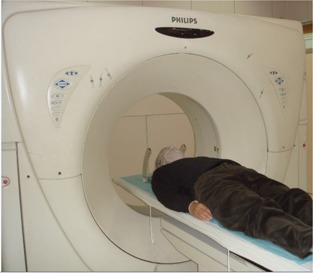
Schematic illustration of the immobilization of patient during CT scan.

The CT scans were obtained on a Philips AcQSim CT simulator (Philips Medical Systems, Cleveland, OH) with IV contrast fluid (Omnipaque). The CT imaging parameters were 140 kVp, 146 mA, matrix 512×512 and FOV of 256 mm, standard head reconstruction kernel. The reconstructed slice thickness was 3 mm. During CT scan, patients were immobilized in the supine position with a thermoplastic face mask (Fig. 1).

The MR images were acquired with a GE 1.5 Tesla unit (GE Medical Systems, Waukesha, WI) using a Torso PA body coil. The patients were immobilized in supine position with the same thermoplastic mask as that used during CT scan. T1‐weighted (repetition time [TR] 300–400 ms and echo time [TE] 10–15 ms) and T2‐weighted (TR 4000–5000 ms and TE 80–100 ms) fast spin‐echo images in the axial plane were obtained with the matrix of 256×256 and FOV of 256 mm. The image slice thickness was 5 mm with a 1‐mm interslice gap.

### C. CT and MRI Registration

The fusion of CT and T2 weighted MR image sets was performed. Each user must finish CT/ MRI registration using the following three registration algorithms, mark‐and‐link, interactive, and Normalized Mutual Information (NMI), separately.

“Mark‐and‐link” registration displays CT and MR image sets side by side on the screen. We identify at least three noncoplanar pairs of matching (conjugate) location points within the two image sets, such as the top of dens axis, the internal carotid artery, the basal artery, the post‐mandibular vein, and the inner auditory canal. Alternating between the two image sets, we will mark conjugate points and link them together.

The interactive registration method displays CT and MR images, one overlaid on the other, in each of four separate image viewports: oblique, axial, sagittal and coronal. CT is gray scale and MRI is in color. We can use any of the normal image manipulation methods: rotation, movement (left/right/up/down), panning left and right, and zooming in or out, to manipulate the MR image to best match with CT.

NMI matching uses the concept of relative entropy between two image sets, which is a measure of how one image explains the other. It is given by the difference between the sum of the entropies of the individual images at overlap and the joint entropy of the combined images. At alignment, the algorithm tries to maximize the mutual information so that the joint entropy is minimized with respect to the entropy of the overlapping part of the individual images. In other words, it tries to calculate the transformation that makes one image the best possible predictor for the other, within the region of overlap. The technique needs no prior segmentation or preprocessing of the images, and was done automatically.

To compare the time of the registration process, the total time required to select the landmarks and for the algorithm to complete the registration was recorded for each registration method. The rotation of MR images along the x‐, y‐, and z‐axes calculated by the three registration algorithms was also recorded.

### D. Evaluation of the Registration Accuracy

**Figure 2:**
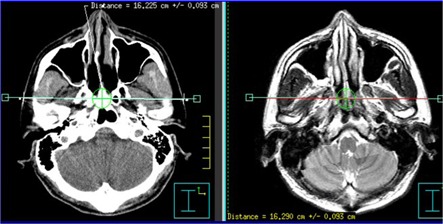
Illustration of measurement of CT/MRI registration error along the x‐ axis. Distance of the skin contour shown on CT is 16.225 cm, and 16.290 cm on MRI; registration error on this slice is 0.065 cm.

**Figure 3:**
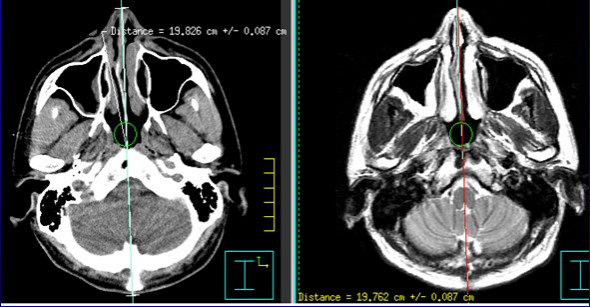
Illustration of measurement of CT/MRI registration error along the y‐axis. Distance of the skin contour shown on CT is 19.826 cm, and 19.762 cm on MRI; registration error on this slice is 0.064 cm.

Registration accuracy was accessed by measuring the difference of the distance along the x‐ (left‐right), and y‐ (anterior‐posterior) axes between the skin contours on CT and MR images, since the skin contour was well visualized on both CT and MR (Fig. 2 and Fig. 3). Measurements were carried out at three transverse levels with 1.5 cm space intervals. The mean value of these measured differences was used to evaluate the registration accuracy along the x‐ and y‐axes. Registration accuracy along the z‐axis was accessed by comparing the z‐coordinate of the three anatomical landmarks: roof of skull bone, bottom of sella turcica, and top of dens axis. The mean value of these differences served as a quantitative measurement of the registration accuracy along the z‐axis. In addition, the contours of the left maxillary sinus were delineated on both CT and the registered MR images, independently. The coordinate differences of the centers of the left maxillary sinus were used as an estimate for registration errors along x‐, y‐ and z‐axes.

All measurements were done by the same doctor to avoid the interobserver variations on the distance measurements. And all measurements were carried out on the ADAC Pinnacle^3^ workstation to which the image data were transferred using self‐developed utility software.

### E. Statistical Analysis

All statistical analyses were performed using the SPSS software (version 10.0). One‐Way ANOVA was used to analyze the difference of the 3 registration methods (ANOVA is short for Analysis of Variance). A two‐tailed *p* value of <0.05 was considered statistically significant in all cases.

## III. RESULTS

### A. Registration Accuracy

The mean distance differences between CT and MR skin contours along the x‐ and y‐axes, and the mean coordinate differences along the z‐axis for three registration algorithms are listed in Table 1. The difference of the fusion accuracy along the x‐axis (F=0.09,p=0.91), y‐axis (F=0.02,p=0.98), and z‐axis (F=0.21,p=0.81) was found to be of no statistical significance.

**Table 1:** Skin contour and coordinate differences between CT and MRI along the x‐, y‐ and z‐axes after registration.

	*Mark‐and‐link (mm)*			*Interactive (mm)*			*NMI (mm)*		
*Case*	*x*	*y*	*z*	*x*	*y*	*z*	*x*	*y*	*z*
1	0.7	1.1	1.2	0.8	1.1	1.1	0.6	1.1	1.0
2	0.9	0.8	0.3	1.1	0.7	0.2	1.0	0.7	0.1
3	0.8	1.3	0.7	0.8	1.3	0.8	0.9	1.3	0.8
4	0.2	1.2	0.4	0.2	1.2	0.3	0.3	1.3	0.5
5	0.4	0.9	1.1	0.4	0.9	0.9	0.5	0.8	1.0
6	0.8	1.3	0.8	0.9	1.2	0.9	0.9	1.3	0.8
7	0.9	1.1	0.6	0.9	1.1	0.5	0.8	1.2	0.5
8	0.7	0.9	0.9	0.8	0.9	0.8	0.7	0.8	0.7
9	0.6	0.9	0.2	0.6	1.0	0.3	0.5	0.9	0.1
10	0.3	1.0	0.0	0.4	1.0	0.8	0.3	1.1	0.5
11	0.8	0.7	0.5	0.8	0.8	0.6	0.8	0.6	0.2
12	0.8	1.1	0.3	0.9	1.3	0.5	0.8	1.2	0.5
Mean	0.66	1.03	0.58	0.70	1.04	0.64	0.68	1.03	0.56

Using the center of the left maxillary sinus as the reference structure for registration errors gave similar results as shown in Table 2. No statistical difference was found among the coordinate differences between CT and MR after registration along the x‐axis (F=0.06,p=0.94), y‐axis (F=0.16,p=0.85), and z‐axis (F=0.03,p=0.97).

**Table 2:** Coordinate differences between CT and MR after registration determined by the center of the left maxillary sinus along x‐, y‐ and z‐axes.

	*Mark‐and‐link (mm)*			*Interactive (mm)*			*NMI (mm)*		
*Case*	*x*	*y*	*z*	*x*	*y*	*z*	*x*	*y*	*z*
1	0.2	0.1	0.2	0.1	0.2	0.3	0.1	0.1	0.2
2	0.1	0.1	0.3	0.1	0.2	0.2	0.1	0.2	0.2
3	0.3	0.3	0.4	0.4	0.3	0.3	0.3	0.3	0.4
4	0.2	0.2	0.4	0.2	0.2	0.3	0.3	0.3	0.5
5	0.4	0.5	0.6	0.4	0.6	0.5	0.5	0.6	0.5
6	0.8	1.0	0.2	0.9	1.1	0.3	0.9	1.1	0.2
7	0.0	0.1	0.6	0.0	0.1	0.5	0.1	0.2	0.5
8	0.4	0.9	0.0	0.5	0.9	0.0	0.6	0.8	0.0
9	0.6	0.5	0.2	0.6	0.6	0.3	0.5	0.7	0.1
10	0.3	1.0	0.0	0.4	1.0	0.2	0.3	1.1	0.1
11	0.8	0.5	0.0	0.8	0.6	0.0	0.8	0.6	0.0
12	0.5	0.5	0.3	0.6	0.6	0.5	0.5	0.6	0.5
Mean	0.38	0.47	0.27	0.42	0.53	0.28	0.42	0.55	0.28

### B. Efficiency of CT and MRI Registration

The time needed to complete acceptable CT and MRI registration using mark‐and‐link, interactive, and NMI fusion algorithms was 6.25±1.27 min,5.25±1.01 min, and 5.15±0.86 min, respectively. More time is needed for the mark‐and‐link method (F=8.74,p=0.001); however, no statistical difference was found between the interactive and NMI methods (p=0.77).

### C. Rotation of MRI to Complete CT and MRI Registration

The angle of rotation of MR image data set along the x‐, y‐, and z‐axes after completing CT and MRI fusion with the three registration algorithms was shown in Table 3. No statistical difference was found in the rotation angles along the x‐axis (*F*=0.01, *p*=0.99), y‐axis (*F*=0.02, *p*=0.98), and z‐axis (*F*=0.02, *p*=0.98).

**Table 3:** Rotation of MR image along the x‐, y‐ and z‐axes after CT and MRI registration.

	*Mark‐and‐link (°)*			*Interactive (°)*			*NMI (°)*		
*Case*	*α*	*β*	*γ*	*α*	*β*	*γ*	*α*	*β*	*γ*
1	1.2	0.7	0.6	1.2	0.6	0.6	1.3	0.8	0.5
2	0.9	1.3	0.9	0.8	1.2	0.9	0.9	1.2	1.0
3	1.9	1.4	1.1	1.8	1.5	1.2	1.9	1.4	1.1
4	0.8	0.9	0.7	0.8	0.8	0.7	0.8	0.8	0.7
5	0.9	1.7	1.2	0.8	1.6	1.1	0.9	1.7	1.2
6	1.1	1.1	0.9	1.3	1.1	0.8	1.1	1.2	0.9
7	0.4	0.7	1.3	0.4	0.7	1.3	0.4	0.8	1.2
8	0.7	0.9	0.7	0.6	0.8	0.7	0.7	0.9	0.7
9	0.8	0.5	1.4	0.8	0.6	1.4	0.7	0.6	1.5
10	0.6	1.3	1.0	0.6	1.2	1.0	0.6	1.4	1.1
11	1.0	1.6	0.8	1.1	1.5	0.8	1.0	1.7	0.7
12	1.3	1.1	1.6	1.4	1.2	1.6	1.2	1.0	1.5
Mean	.98	1.09	1.02	0.97	1.10	1.04	0.96	1.13	1.05

Note: *α, β, γ* refer to the rotation of MR image along the x‐, y‐, and z‐axes, respectively.

## IV. DISCUSSION

The spatial information obtained with different imaging studies can be integrated into one data set with the image registration procedure. A number of registration algorithms have been described in the literature.^(^ ^10^ ^–^ ^16^ ^)^ A stereotactic fixation frame may be attached to the skull for subsequent CT and MR scanning procedures and serve as a reference structure for image registration. Although it can provide fairly reliable registration, it requires a controlled clinical setting, and the image registration can only be carried out prospectively.^(1)^ This disadvantage can be overcome by the so‐called retrospective registration methods, which pose less logistical problems and may be more useful for clinical applications. These registration algorithms were described in detail and, in general, were able to provide satisfactory results.^(^ ^10^ ^–^ ^16^ ^)^ Median errors for CT‐MR registration were in the range of 0.7 mm to 6.3 mm.^(17)^

Some of these retrospective methods such as mark‐and‐link and surface matching required the identification or delineation of several corresponding structures in subsequent image data sets to perform registration.^(10)^ ^,^ ^(11)^ In general, they provide adequate image registration results; however, these methods suffer from the intensive user interaction that is required. As a consequence, the process is highly user‐dependent and relies on the skill of the user. Our study showed that mark‐and‐link matching has failed quite often in the first attempt, and requires several iterations, and hence time, to minimize the residual registration error. An alternative approach is the automated registration technique using intensity matching (Normalized Mutual Information), which has shown to be the best option in terms of both accuracy and efficiency. Regardless of the skill of the user in aligning the landmarks, NMI consistently results in more accurate registration in much less time than that spent by an experienced user in the manual process of image registration.^(^ ^13^ ^–^ ^16^ ^)^

The validation and comparison of registration techniques are impeded by the lack of a “gold standard” for registration tests. West et al.^(17)^ evaluated 16 registration techniques for CT and MR images in a collaborative study of 12 centers. The resultant registrations of all centers were compared with a standard image set, which consisted of a prospective registration technique employing fiducial markers. Median errors for CT‐MR registration were in the range of 0.7 mm to 6.3 mm. Veninga et al.^(1)^ validated NMI method for registration of CT and MRI in 15 brain tumor cases by performing statistical analysis of coordinate differences between CT and MR anatomical landmarks along the x‐, y‐ and z‐axes. The mean coordinate differences between CT and MR landmarks were typically within 0.5 mm along the x‐ and y‐axes, and within 1.0 mm along the z‐axis. Moore et al.^(18)^ employed a head and neck phantom to test the accuracy of CT and MRI registration, and found that the mean difference between the coordinates of the center of shape was 0.43 mm along the x‐axis, and 0.37 mm along the y‐axis. Gedat et al.^(19)^ used both phantom and volunteers to analyze the accuracy of retrospective CT/MRI registration, and reported that the accuracy was 0.0 mm±1.2 mm and 0.2°±0.9° (mean±SD) in phantom experiments, and 0.1 mm±1.5 mm and 0.2°±1.5° in volunteers. In our study, the patients were immobilized with a thermoplastic mask to receive CT and MRI scan. Our results revealed a fairly good immobilization consistency as far as rotations are concerned – typically ≤1.0 degree as indicated by this image registration algorithm evaluation study. We compared the accuracy of manual (mark‐and‐link, interactive) and automated (NMI) registration methods for CT and MR images in 12 NPC patients by using the distance differences of the skin contour and the coordinate differences of anatomical landmarks on the corresponding fused image slices. We also compared the total time required by each registration algorithm to generate the satisfactory results, including the time taken by the physician to align the landmarks in manual registration. We found that the three methods provided similar registration accuracy, which was less than 0.8 mm along the x‐axis, less than 1.1 mm along the y‐axis, and less than 0.7 mm along the z‐axis. But it required more time using the mark‐ and‐link method.

The dominant factors which determine the registration residual errors are: identification of the initial landmarks on each image set during the registration process, accuracy of the registration algorithm, and subjective analysis of the registration results. The centers of the right and left eye globe were used in evaluating the registration errors in 1D.^(1)^ In our opinion, due to the long scan time, it is impossible for the eyes to remain in a fixed position and, furthermore, the eyes are located above the nasopharynx along the z‐axis. The maxillary sinus, on the contrary, remains in a fixed position and is located at the same level as the nasopharynx along the z‐axis. Thus the center of the maxillary sinus was used for analyzing the registration errors in 1D in our study. Registration accuracy can be affected by several other factors as well, such as image distortion,^(20)^ body position, and patient motion during the image acquisition.^(15)^ In our study, patients were immobilized with the same thermoplastic mask for both CT and MR scans, which ensured the same body position and minimized the patient motion during MR scan.

In this study, we did not measure the extent of system‐related distortions or object‐induced distortions in MRI. However, based on our measurements, the CT and MRI registration errors were very small along the x‐, y‐, and z‐axes, which would indicate that the system‐ and object‐induced distortions are negligible.

## V. CONCLUSIONS

Mark‐and‐link, interactive, and NMI methods can provide excellent registration accuracy as evaluated by the measurement of coordinate differences between a series of well‐defined landmarks in CT and MR data sets. But more time is required for the mark‐and‐link method.

## References

[1] Veninga T , Huisman H , van der Maazen RW , Huizenga H . Clinical validation of the normalized mutual information method for registration of CT and MR images in radiotherapy of brain tumors. J Appl Clin Med Phys. 2004;5(3):66–79. 15753941 10.1120/jacmp.v5i3.1959 PMC5723487

[2] Fraass BA , McShan DL , Diaz RF , et al. Integration of magnetic resonance imaging into radiation therapy treatment planning: I. Technical consideration. Int J Radiat Oncol Biol Phys. 1987;13(12):1897–1908. 3679929 10.1016/0360-3016(87)90358-0

[3] Khoo VS , Dearnaley DP , Finnigan DJ , Padhani A , Tanner SF , Leach MO . Magnetic resonance imaging (MRI): considerations and applications in radiotherapy treatment planning. Radiother Oncol. 1997;42(1):1–15. 9132820 10.1016/s0167-8140(96)01866-x

[4] Yanke BR , Ten Haken RK , Aisen A , Fraass BA , Thornton Jr AF . Design of MRI scan protocols for use in 3‐D, CT based treatment planning. Med Dosim. 1991;16(4):205–11. 1764171 10.1016/0958-3947(91)90084-f

[5] Sailer SL , Rosenman JG , Soltys M , Soltys M , Cullip TJ , Chen J . Improving treatment planning accuracy through multimodality imaging. Int J Radiat Oncol Biol Phys. 1996;35(1):117–24. 8641907 10.1016/s0360-3016(96)85019-x

[6] Aoyama H , Shirato H , Nishioka T , et al. Magnetic resonance imaging system for three‐dimensional conformal radiotherapy and its impact on gross tumor volume delineation of central nervous system tumors. Int J Radiat Oncol Biol Phys. 2001;50(3):821–27. 11395252 10.1016/s0360-3016(01)01598-x

[7] Khoo VS , Adams EJ , Saran F , et al. A comparison of clinical target volumes determined by CT and MRI for the radiotherapy planning of base of skull meningiomas. Int J Radiat Oncol Biol Phys. 2000;46(5):1309–17. 10725645 10.1016/s0360-3016(99)00541-6

[8] Nishioka T , Shiga T , Shirato H , et al. Image fusion between 18FDG‐ PET and MRI/CT for radiotherapy planning of oropharyngeal and nasopharyngeal carcinomas. Int J Radiat Oncol Biol Phys. 2002;53(4):1051–57. 12095574 10.1016/s0360-3016(02)02854-7

[9] Emami B , Sethi A , Petruzzelli GJ . Influence of MRI on target volume delineation and IMRT planning in nasopharyngeal carcinoma. Int J Radiat Oncol Biol Phys. 2003;57(2):481–88. 12957260 10.1016/s0360-3016(03)00570-4

[10] Hill DLG , Hawkes DJ , Crossman JE , et al. Registration of MR and CT images for skull base surgery using point‐like anatomical features. Br J Radiol. 1991;64(767):1030–35. 1742584 10.1259/0007-1285-64-767-1030

[11] Jiang H , Robb RA , Holton KS . A new approach to 3‐D registration of multimodality medical images by surface matching. Proc SPIE 1992;1808:196–213.

[12] Huizenga H , Levendag PC , De Porre PM , Visser AG . Accuracy in radiation field alignment in head and neck cancer: A prospective study. Radiother Oncol. 1988;11(2):181–87. 3353522 10.1016/0167-8140(88)90254-x

[13] Van den Elsen PA , Pol EJD , Sumanaweera TS , Hemler PF , Napel S , Adler JR . Grey value correlation techniques used for automatic matching of CT and MR brain and spine images. Proc SPIE 1994;2359:227–37.

[14] Studholme C , Hill DLG , Hawkes DJ . An overlap invariant entropy measure of 3D medical image alignment. Pattern Recog. 1990;32:71–86.

[15] Studholme C , Hill DLG , Hawkes DJ . Automated 3‐D registration of MR and CT images of the head. Med Image Anal. 1996;1(2):163–75. 9873927 10.1016/s1361-8415(96)80011-9

[16] Sarkar A , Santiago RJ , Smith R , Kassaee A . Comparison of manual vs. automated multimodality (CT‐MRI) image registration for brain tumors. Med Dosim. 2005;30(1):20–24. 15749007 10.1016/j.meddos.2004.10.004

[17] West J , Fitzpatrick JM , Wang MY , et al. Comparison and evaluation of retrospective intermodality brain image registration techniques. J Comput Assist Tomogr. 1997;21(4):554–66. 9216759 10.1097/00004728-199707000-00007

[18] Moore CS , Liney GP , Beavis AW . Quality assurance of registration of CT and MRI data sets for treatment planning of radiotherapy for head and neck cancers. J Appl Clin Med Phys. 2004;5(1):25–35. 15753931 10.1120/jacmp.v5i1.1951 PMC5723443

[19] Gedat E , Braun J , Sack I , Bernarding J . Prospective registration of human head magnetic resonance images for reproducible slice positioning using localizer images. J Magn Reson Imaging. 2004;20(4):581–87. 15390147 10.1002/jmri.20153

[20] Maurer Jr CR , Aboutanos GB , Dawant BM , et al. Effect of geometrical distortion correction in MR on image registration accuracy. J Comput Assist Tomogr. 1996;20(4):666–79. 8708077 10.1097/00004728-199607000-00032

